# Impaired GABAergic regulation and developmental immaturity in interneurons derived from the medial ganglionic eminence in the tuberous sclerosis complex

**DOI:** 10.1007/s00401-024-02737-7

**Published:** 2024-05-07

**Authors:** Mirte Scheper, Frederik N. F. Sørensen, Gabriele Ruffolo, Alessandro Gaeta, Lilian J. Lissner, Jasper J. Anink, Irina Korshunova, Floor E. Jansen, Kate Riney, Wim van Hecke, Angelika Mühlebner, Konstantin Khodosevich, Dirk Schubert, Eleonora Palma, James D. Mills, Eleonora Aronica

**Affiliations:** 1 Department of (Neuro)Pathology, Amsterdam Neuroscience, Amsterdam UMC Location University of Amsterdam, Meibergdreef 9, Amsterdam, The Netherlands; 2 Biotech Research and Innovation Centre (BRIC), Faculty of Health and Medical Sciences, University of Copenhagen, 2200 Copenhagen, Denmark; 3 Department of Physiology and Pharmacology, University of Rome Sapienza, 00185 Rome, Italy; 4 UCL Queen Square Institute of Neurology, London, WC1N 3BG UK; 5 Department of Child Neurology, Brain Center University Medical Center, Member of ERN EpiCare, 3584 BA Utrecht, The Netherlands; 6 Faculty of Medicine, The University of Queensland, St Lucia, QLD 4067 Australia; 7 Neurosciences Unit, Queensland Children’s Hospital, South Brisbane, QLD 4101 Australia; 8 Department of Pathology, University Medical Center Utrecht, Utrecht, The Netherlands; 9 Department of Cognitive Neurosciences, Radboudumc, Donders Institute for Brain Cognition and Behaviour, 6525 HR Nijmegen, The Netherlands; 10 IRCCS San Raffaele Roma, 00163 Rome, Italy; 11 Chalfont Centre for Epilepsy, Bucks, SL9 0RJ UK; 12 Stichting Epilepsie Instellingen Nederland (SEIN), Heemstede, The Netherlands

**Keywords:** GABAergic interneurons, snRNA-seq, Ganglionic eminence, Somatostatin, Immaturity

## Abstract

GABAergic interneurons play a critical role in maintaining neural circuit balance, excitation–inhibition regulation, and cognitive function modulation. In tuberous sclerosis complex (TSC), GABAergic neuron dysfunction contributes to disrupted network activity and associated neurological symptoms, assumingly in a cell type-specific manner. This GABAergic centric study focuses on identifying specific interneuron subpopulations within TSC, emphasizing the unique characteristics of medial ganglionic eminence (MGE)- and caudal ganglionic eminence (CGE)-derived interneurons. Using single-nuclei RNA sequencing in TSC patient material, we identify somatostatin-expressing (SST+) interneurons as a unique and immature subpopulation in TSC. The disrupted maturation of SST+ interneurons may undergo an incomplete switch from excitatory to inhibitory GABAergic signaling during development, resulting in reduced inhibitory properties. Notably, this study reveals markers of immaturity specifically in SST+ interneurons, including an abnormal *NKCC1/KCC2* ratio, indicating an imbalance in chloride homeostasis crucial for the postsynaptic consequences of GABAergic signaling as well as the downregulation of GABA_A_ receptor subunits, *GABRA1*, and upregulation of *GABRA2.* Further exploration of SST+ interneurons revealed altered localization patterns of SST+ interneurons in TSC brain tissue, concentrated in deeper cortical layers, possibly linked to cortical dyslamination. In the epilepsy context, our research underscores the diverse cell type-specific roles of GABAergic interneurons in shaping seizures, advocating for precise therapeutic considerations. Moreover, this study illuminates the potential contribution of SST+ interneurons to TSC pathophysiology, offering insights for targeted therapeutic interventions.

**Supplementary Information:**

The online version contains supplementary material available at 10.1007/s00401-024-02737-7.

## Introduction

Tuberous sclerosis complex (TSC) is a multisystem genetic disorder that is characterized by age-related development of lesions in the brain, skin, heart, kidneys, and other organs. Clinically, the TSC phenotype is characterized by a spectrum of symptoms including epilepsy and neuropsychiatric disorders [11, 43]. TSC is caused by loss-of-function mutations in either the *TSC1* or *TSC2* gene, encoding for hamartin and tuberin, respectively. A loss-of-function mutation in *TSC1* or *TSC2* leads to disruption of the TSC1–TSC2 complex, inducing hyperactivity of the mammalian target of rapamycin (mTOR) pathway [11].

While overactivation of the mTOR pathway is known to be associated with both genetic and acquired epilepsies [23, 40, 41, 59], individuals with TSC may additionally present with a spectrum of different neuropsychiatric co-morbidities known as tuberous sclerosis-associated neuropsychiatric disorders (TAND) [12, 62]. These TAND can manifest in various domains, affecting behavioral aspects such as sleep disturbances and aggression, psychiatric aspects including autism spectrum disorder (ASD) (present in 40–50% of patients) and attention deficit hyperactivity disorder (ADHD) (30–50%), intellectual disability (ID) (seen in 40–50% of patients), and learning disorders [12, 62]. It is important to note that individuals with TSC often experience a complex interplay of these manifestations, with many exhibiting multiple and overlapping TAND simultaneously, further complicating and increasing disease burden and necessitating a comprehensive and individualized approach to management and intervention.

Accumulating evidence suggests that dysregulated GABAergic signaling could result in both epilepsy and neuropsychiatric disorders. Both the delay and lasting impairment of the maturation of GABAergic signaling play a role in this. GABAergic signaling immaturity, characterized by the altered expression of GABA_A_-receptor subunits and cation-chloride cotransporters, disrupts the normal maturation of the postsynaptic consequences of GABAergic signaling [2, 10, 22, 24, 53, 58]. On the other hand, the impaired development, migration, or dysfunction of cortical GABAergic interneurons, referred to as interneuronopathy, further exacerbates the dysregulation of GABAergic network formation and related signaling and contribute to the large variety of epilepsies and co-occurring neurodevelopmental disorders (NDDs) [27, 39, 50]. GABAergic signaling is mediated by a variety of different interneuron subpopulations [60, 66]. The neuronal diversity in the cortex is associated with the specific transient germinal zones, named the medial and caudal ganglionic eminences (MGE and CGE, respectively) [38, 42]. These eminences give rise to distinct interneuron subpopulations, contributing to the complexity of cortical neuronal diversity. The MGE gives rise to parvalbumin (*PVALB*)-expressing cortical interneurons, somatostatin (*SST*)-expressing cortical interneurons, and a selection of neuropeptide Y (*NPY*)-expressing interneurons [28]. In contrast, the CGE gives rise to VIP-expressing cortical interneurons, reelin-expressing cortical interneurons, and other cortical interneurons [37].

Moreover, many studies have looked at the expression of GABA-related genes in ASD and have shown that reductions in both *GAD65/67* and GABA_A_ receptor subunit expression can be found in postmortem samples of ASD patients [14, 15]. In line with this finding, GABA_A_ receptor subunit α1 expression was found to be decreased in tissue resected from individuals with TSC [53, 58]. Other studies have shown that downregulation of the α1 subunit precedes epileptogenesis and the overexpression of α1 can reduce the occurrence of spontaneous seizures by 60% in mice [7, 51]. Furthermore, inhibitory synaptic signaling demonstrates both temporal and region-dependent changes when overactivity of the mTOR pathway occurs in mice [4]. Interestingly, selective deletion of *Tsc1* in MGE-derived interneurons increased mTOR activity, but failed to affect the amplitudes or frequency of GABAergic postsynaptic currents [35].

Given the broad phenotypic heterogeneity of TSC and the wide spectrum of TAND, it is plausible to propose that functional alteration in neuronal activity during brain development may contribute to the complex disease manifestations. Alterations in the excitatory/inhibitory (E/I) balance in the brain has gained significant attention. Previous studies have provided evidence that GABAergic signaling is reduced in TSC and that increased activity in the mTOR pathway can lead to a cascade of induced changes and possible consequent adjustments, resulting in the overexcitability of the brain network [25–28]. Taking into consideration the observation that GABAergic neurons play a role in neuronal excitation during early development, it is conceivable that in individuals with TSC inhibition is reduced, leading to an incomplete transition into the inhibitory phenotype and the presence of immature interneurons [53]. However, the specific alterations within interneuron subpopulations in TSC remain largely unexplored. It is now recognized that interneurons can be categorized into distinct subtypes based on their molecular and functional properties [17]. This raises the intriguing question of whether specific interneuron subpopulations within TSC display unique alterations or if all subpopulations are uniformly affected.

Therefore, our objective was to determine whether we could identify specific GABAergic dysregulation based on the origin and specific phenotype of interneurons. We performed single nuclei RNA sequencing (snRNAseq) which provided the opportunity to specifically look at the interneurons of control and TSC samples. We analyzed the snRNAseq data and further aimed to investigate the expression of different GABA-related genes, including GABA_A_ receptor subunits. Given the knowledge on the immaturity and development of interneurons we mainly focus on the α1 and α2 subunits, along with chloride transporter sodium–potassium–chloride cotransporter 1 (*NKCC1*) and potassium–chloride cotransporter 2 (*KCC2*). Moreover, we investigated the localization of SST+ interneurons by immunohistochemistry and voltage-clamp recordings in *Xenopus* oocytes were used to determine functional immaturity of GABAergic signaling in TSC tissue.

## Materials and methods

### Dissection of the frontal cortex from frozen tissue

Surgical and postmortem brain tissues were selected from the archives of the Departments of Neuropathology of the Amsterdam UMC (Amsterdam, The Netherlands), the UMC Utrecht (Utrecht, The Netherlands), and Queensland Children’s Hospital (Brisbane, Australia). For single-nuclei RNA sequencing, cortical brain samples from individuals diagnosed with TSC (*n* = 11) were obtained from brain surgery for intractable epilepsy. The resected tissue is characterized by histologically dysplastic features, specifically identified as TSC tuberal tissue (TSC-cortical tuber). Informed consent was acquired for the use of brain tissue for research purposes. Control samples (*n* = 6) were obtained at autopsy from age-matched controls, without a history of seizures or other neurological diseases. All autopsies were performed within 9 h after death. All samples had an RNA integrity number of > 5. For all controls, we included the anterior prefrontal cortex or middle frontal area (depending on the availability). For *Xenopus* oocyte experiments, we used five additional resected samples of TSC. For control samples in these experiments, careful analysis and evaluation of clinical data were used to include samples that displayed normal cortical structure and no significant brain pathology. Tissue was obtained and used in accordance with the Declaration of Helsinki and the Amsterdam UMC Research Code provided by the Medical Ethics Committee and according to the Amsterdam UMC and UMC Utrecht Biobank Regulations (W21-295; 21-174). Clinical information about the brain samples is summarized in supplementary Table 1.

### Single nucleus RNA-seq (snRNA-seq)

#### Nuclei extraction and FACS sorting

Nuclei extraction and fluorescence-activated cell sorting (FACS) was performed as described in detail before [5, 30, 48]. The tissue samples, including both TSC and control samples, were processed in parallel whenever possible. Tissue was removed from -80 °C and transferred to a chilled homogenization buffer and homogenized. The resulting homogenate was filtered through a 40-μm cell strainer and centrifuged at 1000*×g* for 8 min at 4 °C. The supernatant was removed and the pellet resuspended in 250 µl 0.5% bovine serum albumin (BSA) in 1X PBS with RNAse inhibitor (Takara, 2313B, final concentration 0.4 U/μL) for blocking and incubated for 15 min on ice. Subsequently, samples were stained with anti-NeuN antibody Ms-NeuN-488 (Millipore, MAB3777x, 1ug/µL, 1:1890) and incubated in the dark for 10 min at 4 °C. Afterward, the suspensions were centrifuged at 1000*×g* for 8 min at 4 °C, and the pellets were resuspended and filtered through 35 µM strainers into FACS tubes resulting in a final volume of 500 µL. To gate for nuclei, 0.75 µL of 7-aminoactinomycin (7-AAD), a nucleic acid chelating fluorophore, was added to samples on ice. Immediately after that, FACS was performed and NeuN-positive cells were enriched and sorted into BSA pre-coated 1.5 mL LoBind Eppendorf tubes at 4 °C, and 20% of negative NeuN fraction was added to the enriched fraction to yield the final sample composition (80% NeuN+, 20% NeuN− nuclei).

#### Library preparation and sequencing

RNA-sequencing library preparation and sequencing were also performed as described in detail before [5, 48]. The Chromium Single Cell 3′ Reagent Kits v3.1 from 10 × Genomics were employed for library preparation. The procedure involved counting the nuclei under a microscope and combining them with reverse transcription mix and v3.1 Gel Beads on Chromium Chip G. This mixture was partitioned into gel beads-in-emulsion (GEMs) using the Chromium Controller. Following reverse transcription, the samples were frozen for up to a week. Afterward, up to four samples from different 10 × runs were processed together for cDNA cleanup and preamplification. The cDNA was then quantified on the Qubit HS dsDNA Assay Kit (Thermo Fisher Scientific, Q32854), Qubit Fluorometer and High Sensitivity DNA Kit (Agilent, 5067-4626) and Agilent 2100 Bioanalyzer and the same quantity was used for fragmentation, end repair, and A-tailing. Fragments were cleaned up, and subsequent steps included adapter ligation, cleanup, and sample index PCR. The libraries were cleaned up, quantified using the Agilent 2100 Bioanalyzer system, and pooled based on the expected number of nuclei per sample. Finally, the libraries were sequenced on two 100 cycle NovaSeq 6000 S2 flow cells (Illumina, 20012861) using an Illumina NovaSeq 6000 (Illumina, 20012850).

#### Data processing

After pre-processing, including CellBender 0.2.2 and filtering of the data, Seurat (v.4.1.3) was used to further process the data, following the guidelines for snRNA-seq data [21]. All steps used default options unless stated otherwise. For each sample, an expression matrix containing unique molecular identifiers (UMIs) per nucleus per gene was imported as a 10 × data object. Only nuclei with more than 200 genes and less than 5% of genes originating from mitochondrial sources were retained. Data was then imported as a Seurat object and all samples were integrated using the FindIntegrationAnchors and IntegrateData functions. The count matrix was scaled and normalized by variance stabilizing transformation (VST) with Seurat’s ScaleData and NormalizeData commands, respectively. The 2000 most variable features were then selected with the FindVariableFeatures command for the principal component analysis (PCA), which was performed by the RunPCA command. The PCs generated by the PCA were assessed with ElbowPlot and JackStraw analyses by using up to 20 different components. The resulting PCs were used for Jaccard-weighted, shared nearest neighbor (SNN) distance calculations and graph generation. The graph was then subjected to Louvain clustering and uniform manifold approximation and projection (UMAP) for dimension reduction to visualize nuclear transcriptomic profiles in two-dimensional space. After changing the default assay of the dataset from integrated to RNA, a set list of marker genes (Supplementary Table 2) was used to annotate the found cell clusters. From here, GABAergic interneurons were extracted from the dataset and used for further analysis.

#### Pseudo-bulk differential expression

To perform differential expression analysis between control and TSC samples, we performed pseudo-bulk analysis. This approach involves aggregating cells within each biological sample to create 'pseudo-bulks'. This aggregation is essential because single cells within the same biological sample are not independent of each other. Specifically, we aggregated GABAergic interneuron counts for each sample and generated a corresponding metadata column. Differential expression analysis was performed using the R package DESeq2 [33]. To control the false discovery rate, we applied the Benjamini–Hochberg correction, considering gene expression changes with an adjusted *p* value < 0.05 as statistically significant. Subsequently, we visualized differentially expressed genes through volcano plots.

### Immunohistochemistry

Human brain tissue fixed in 10% buffered formalin and embedded in paraffin was mounted on pre‐coated glass slides (Star Frost, Waldemar Knittel, Braunschweig, Germany). Sections were deparaffinized in xylene and rinsed in ethanol (100, 100, 96%). Antigen retrieval was performed using a pressure cooker in 0.01 M sodium citrate buffer (pH 6.0) at 120 °C for 10 min. Slides were cooled in ice water for 15 min, washed with phosphate‐buffered saline (PBS, pH 7.4) and incubated for 1 h at RT with a primary antibody against SST (1:300, mouse monoclonal Ab, SantaCruz, SC-55565). Sections were washed and incubated for 30 min at RT with Brightvision poly‐alkaline phosphatase (AP) anti‐mouse secondary antibody (Immunologic, Duiven, the Netherlands). Then, sections were again washed with PBS and AP activity was visualized with the AP substrate kit III Vector Red (SK‐5100, Vector Laboratories Inc., Burlingame, CA, USA). Subsequently, sections were cooked in a sodium citrate buffer and then washed with PBS. Incubation with FOXP2 antibody (1:200, rabbit polyclonal Ab, Atlas Antibodies, HPA000382) was performed at 4 °C overnight in antibody diluent. The next day, sections were washed with PBS and incubated with Brightvision poly‐alkaline phosphatase (AP) anti‐rabbit (Immunologic, Duiven, the Netherlands) for 30 min at room temperature and again washed with PBS. AP activity was visualized with the AP substrate kit III Vector Blue (SK‐5300, Vector Laboratories Inc., Burlingame, CA, USA). The development process was monitored, and the reaction was stopped by washing the samples when the desired signal intensity was achieved. Sections were then dried and coverslipped.

#### Immunohistochemical quantification

Stainings of FOXP2 and SST were quantified by counting the number of SST-positive cells in layer 2/3/4 and 5/6 separately using ImageJ. FOXP2 staining was used as reference for cortical layer 5/6. For each case, four representative images were taken (2× magnification) for control (*n* = 3) and TSC (*n* = 3) in which all cortical layers were visible. Finally, a ratio was calculated by dividing the SST-positive cell count in layer 2/3/4 by the cell count in layer 5/6 in each image.

### *Xenopus* oocytes

#### Membrane preparation and oocytes’ injection

The preparation of human membranes, the cytoplasmic injection in *Xenopus laevis* oocytes and the electrophysiological recordings of GABA currents were executed as previously described [1, 44]. These membranes are extracted from control and pathological tissues and injected in the cytoplasm of the oocytes. The transplanted receptors maintain their native characteristics (see also [45]). In another set of experiments, we performed intranuclear injection in *Xenopus* oocytes of human complementary DNA (cDNAs) encoding for α2, β2 and γ2 GABA_A_ subunits (pcDNA3 vector) in two different ratios (1:1:1 vs 3:1:1) [53]. Human α2β2γ2 cDNA was provided as a gift by Dr. Keith Wafford. The use of female *X. laevis* frogs and the surgical methods for oocyte extraction and for their use conformed to the Italian Ministry of Health guidelines (authorization no. 427/2020-PR).

#### Electrophysiological recordings

The electrophysiological experiments with the microtransplanted oocytes were performed 24–48 h from the cytoplasmatic injection using the ‘two-electrode voltage-clamp’ technique. At controlled room temperature ranging from 21 to 23 °C, the oocytes were placed in a recording chamber (0.1 mL volume) and constantly perfused with oocyte Ringer solution (OR: NaCl 82.5 mM; KCl 2.5 mM; CaCl_2_ 2.5 mM; MgCl_2_ 1 mM; Hepes 5 mM, adjusted to pH 7.4 with NaOH), while clamping with two microelectrodes filled with KCl or K^+^acetate 3 M [54]. Neurotransmitter application was digitally regulated by a computer (Biologique RSC-200; Claix, France) using a gravity-driven multi-valve perfusion system (8–10 mL/min) to ensure the exact duration of each application. GABA (250 μM unless otherwise specified) was applied for 4 s to oocytes to elicit inward currents (${{\text{I}}}_{{\text{GABA}}}$). In all the experiments, the stability of GABA-evoked currents (I_GABA_) was evaluated through two consecutive neurotransmitter applications, separated by a 4 min washout, and only the cells that showed a < 5% variation in current amplitude were used for experiments. GABA was purchased from Tocris Bioscience (Bristol, UK) and dissolved in sterile water and diluted to the desired concentration in OR before each experiment. GABA current reversal potential (E_GABA_) was calculated by a current–voltage (I–V) relationship. To obtain the E_GABA_, we held the oocytes at −60 mV and stepped the membrane potential for a few minutes at the desired value before neurotransmitter application. Then, the I–V relationships were fitted with a linear regression curve-fitting software (Sigmaplot 15). In another set of experiments, we performed dose–response relationships by applying different neurotransmitter concentrations to oocytes held at −60 mV and we calculated the apparent affinity (EC_50_) of GABA by fitting all the data to Hill equations, as previously described [44].

#### Statistical analysis of electrophysiology data

Data are reported as mean ± SEM. Unless otherwise indicated, numbers (n) refer to oocytes used in each experiment. Before data analysis, normal distribution was assessed with Shapiro–Wilk test and, according to the result, parametric (Student's *t* test,) or non-parametric (Wilcoxon signed rank test, Mann–Whitney rank sum test) tests were used and performed with Sigmaplot 15 software. Differences between two data sets were considered significant when *p* < 0.05, two tailed.

## Results

### Expression of interneuron subpopulation markers in TSC

To investigate the GABAergic interneurons in the context of TSC, we initially focused on the expression profiles of specific markers associated with interneuron subpopulations. We utilized the 10 × chromium platform to perform snRNA-seq on nuclei from both human TSC and control brain tissues samples. The resulting data was preprocessed, filtered, and integrated as outlined in “Materials and methods” section.

**Fig. 1:**
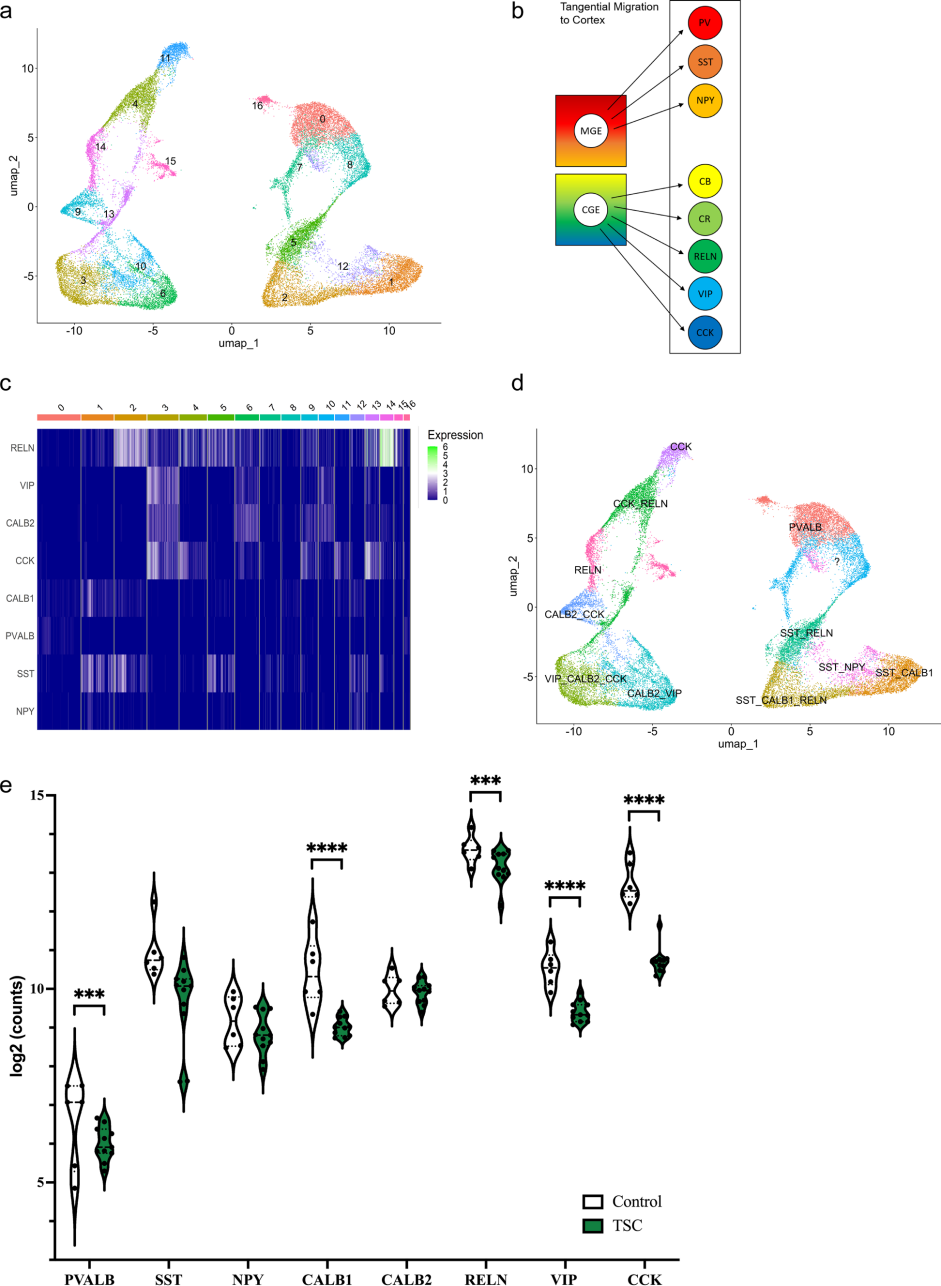
Interneuron subpopulations in the tuberous sclerosis complex (TSC). **a** UMAP clustering analysis based on single-nucleus RNA sequencing (snRNA-seq) data, revealing 17 distinct interneuron clusters. Each cluster is represented by a unique color code, showing the heterogeneity of interneuron subpopulations. **b** Overview of interneuron subpopulations arising from the medial ganglionic eminence (MGE) and caudal ganglionic eminence (CGE). MGE-derived subpopulations include parvalbumin (*PVALB*), somatostatin (*SST*), and neuropeptide Y (*NPY*) interneurons, while CGE-derived subpopulations consist of calbindin (CB; *CALB1*), calretinin (CR; *CALB2*), reelin (*RELN*), vasoactive intestinal peptide (*VIP*), and cholecystokinin (*CCK*) interneurons. **c** Expression matrix illustrating the expression levels of interneuron subpopulations across 17 distinct interneuron clusters. Each row represents a specific subpopulation, and each column represents a distinct interneuron cluster. The color scale indicates the relative expression level, with higher expression shown as white or green. **d** UMAP plot displaying the spatial localization and clustering of interneuron subpopulations. Each subpopulation is indicated by a unique color, facilitating visual identification, and demonstrating their distribution across the clusters. **e** Violin plot depicting the expression levels of *PVALB, SST, NPY, CALB1, CALB2*, *RELN, VIP* and *CCK* in TSC. *PVALB*, *CALB1*, *RELN*, *VIP,* and *CCK* show downregulation in TSC. Statistical significance is denoted as *** (*p* < 0.001), **** (*p* < 0.0001). As each UMAP plot is generated separately, the colors between the plots are not linked

We then annotated GABAergic interneuron clusters by means of glutamate decarboxylase 1 (*GAD1*) and glutamate decarboxylase 2 (*GAD2*), resulting in the identification of 17 distinct interneuron clusters, none of which were specific to TSC (Fig. 1a). In a next step, we distinguished between specific classes of GABAergic interneurons by investigating the expression of interneuron subpopulation markers. The medial ganglionic eminence (MGE) is known to produce mostly parvalbumin (*PVALB*), somatostatin (*SST*), and neuropeptide Y (*NPY*) positive interneurons, whereas cholecystokinin (*CCK*), vasoactive intestinal peptide (*VIP*), reelin (*RELN*), calbindin (CB; *CALB1*), and calretinin (CR; *CALB2*)-positive interneurons originate in the caudal ganglionic eminence (CGE) (Fig. 1b). We examined the expression of these markers in each cluster using a heatmap for the different types of interneuron subpopulations (Fig. 1c). This allowed us to annotate the different interneuron clusters and show the different interneuron subpopulations in our UMAP (Fig. 1d). Clusters with similar expression profiles were combined, resulting in a lower number of eventual annotated clusters. Additionally, we conducted differential expression (DE) analysis using a pseudo-bulk approach to compare the expression profiles between individuals with TSC and control. We found a significant downregulation of *PVALB* expression, as well as *CALB1*, *RELN*, *VIP* and *CCK* (Fig. 1e). These results suggest that there may be alterations in the presence of certain interneuron subpopulations in TSC, which could contribute to the pathophysiology of the disorder.

### Distinctive expression patterns of MGE- and CGE-derived interneurons in snRNA-seq data

**Fig. 2:**
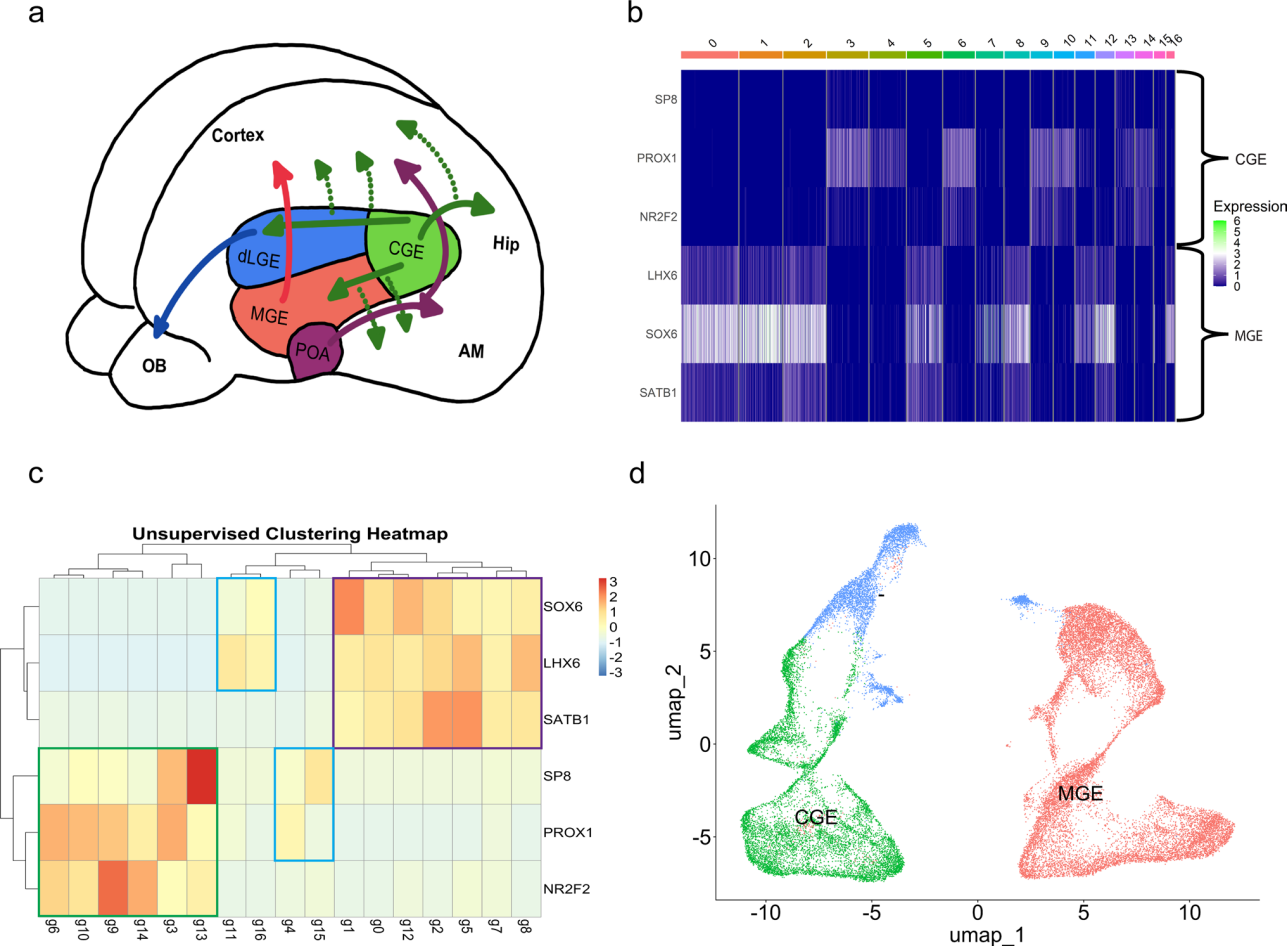
Separation of interneurons from medial ganglionic eminence (MGE) and caudal ganglionic eminence (CGE) in control and TSC human frontal cortex. **a** Schematic overview illustrating the location of the MGE and CGE and their migration patterns during development. **b** Transcription factor analysis highlighting the expression pattern of factors associated with the MGE and CGE. Each row represents a specific transcription factor, and each column represents a distinct interneuron cluster. The color scale indicates the relative expression level, with higher expression shown as white or green. Clear distinctions can be observed, emphasizing the molecular differences between these two interneuron populations. **c** Unsupervised clustering heatmap illustrating the shared expression patterns of MGE- and CGE-derived interneuron subpopulations. The heatmap visually demonstrates the hierarchical clustering of samples based on shared transcriptional signatures. Clusters within the green boxes belong to the CGE-derived interneuron subpopulation. Purple indicates MGE-derived interneuron subpopulations and blue remains undetermined. (Clustering method = complete). **d** UMAP plot displaying the interneuron clusters segregated into CGE and MGE clusters. The separation of interneurons from the MGE and CGE is evident with only two clusters of which it is unsure what their origin is based on the original UMAP combined with the unsupervised clustering based on transcription factors

The medial ganglionic eminence (MGE) and caudal ganglionic eminence (CGE) are two critical regions in the developing brain that give rise to distinct subpopulations of GABAergic interneurons. These interneurons play crucial and specific roles in the regulation of cortical excitability, signal processing and the maintenance of neuronal network dynamics. During brain development, interneurons originating from the MGE and CGE undergo distinct migration patterns to populate specific cortical regions (Fig. 2a) [3]. MGE-derived interneurons predominantly migrate tangentially before switching to radial migration, while CGE-derived interneurons exhibit a radial migration pattern only. This differential migration contributes to the establishment of diverse interneuron subpopulations across the cortical layers. To gain insights into the cellular heterogeneity of the cortex in individuals with TSC, we aimed to characterize interneuron subpopulation and determine whether there are subpopulation-specific differences.

Developmental studies have previously demonstrated distinct differences in transcriptome profiles between interneurons derived from the medial and caudal ganglionic eminence (MGE and CGE, respectively). Notably, within the population of interneurons, two primary clusters were also clearly distinguishable. Transcription factors such as SP8 transcription factor (SP8), prospero homeobox protein 1 (PROX1), and COUP transcription factor 2 (NR2F2) have been found to be predominantly expressed in CGE-derived neurons, while LIM homeobox 6 (LHX6), SRY-box transcription factor 6 (SOX6), and special AT-rich sequence-binding protein-1 (SATB1) have been identified as markers for interneuron clusters originating from the MGE [29]. To determine the origin of interneuron populations in TSC, we examined the expression of these markers in separate interneuron clusters (Fig. 2b). Based on marker expression, we hypothesized that the left cluster containing sub-clusters 0, 1, 2, 5, 7, 6, 12, and 16 consisted of interneuron populations that originated from the MGE, while the remaining sub-clusters on the right originated from the CGE. We performed unsupervised clustering based on expression patterns of SP*8, PROX1, NR2F2, LHX6, SOX6*, and *SATB1* to confirm our hypothesis (Fig. 2c). The analysis excluded four clusters that could not be assigned confidently to either of the two main clusters (Fig. 2d). Consequently, these excluded clusters were not considered in subsequent analyses. The accurate distinction between interneurons derived from the MGE and the CGE is of importance for the analysis of this snRNA-seq data. MGE- and CGE-derived interneurons exhibit distinct molecular profiles, functional properties, and anatomical distributions, contributing to the diverse composition of cortical interneuron subpopulations. Separating these two subpopulations allows us to gain valuable insight into the unique molecular markers and regulatory mechanisms of these subpopulations.

### GABAergic signaling and expression in TSC interneurons

Gamma-aminobutyric acid (GABA) signaling is a fundamental mechanism in the central nervous system (CNS) that regulates neuronal excitability and maintains the balance between excitation and inhibition. In the mature brain GABA acts as the primary inhibitory neurotransmitter, exerting its effects through GABA receptors located on postsynaptic neurons. In the developing brain, GABA signaling undergoes a critical maturation process, transitioning from an excitatory to an inhibitory role [60]. This shift is largely attributed to the dynamic interplay between the activity of two key transporters, the sodium–potassium–chloride cotransporter 1 (*NKCC1*) and the potassium–chloride cotransporter 2 (*KCC2*) [6, 10] regulating the chloride gradient between intra- and extracellular domains. Therefore, we wanted to investigate whether any of the previously found GABAergic interneuron subpopulations had an immature phenotype. For this analysis, we initially divided the interneurons into MGE- and CGE-derived interneurons to understand whether GABA dysregulation is origin specific or a general phenomenon in TSC patients. Analysis on the NKCC1/KCC2 ratio in all MGE- and CGE-derived interneurons taken together, showed no differences. To further investigate possible immaturity, DE was performed in both the MGE and the CGE cluster with a focus on GABA receptor subunit expression.

**Fig. 3:**
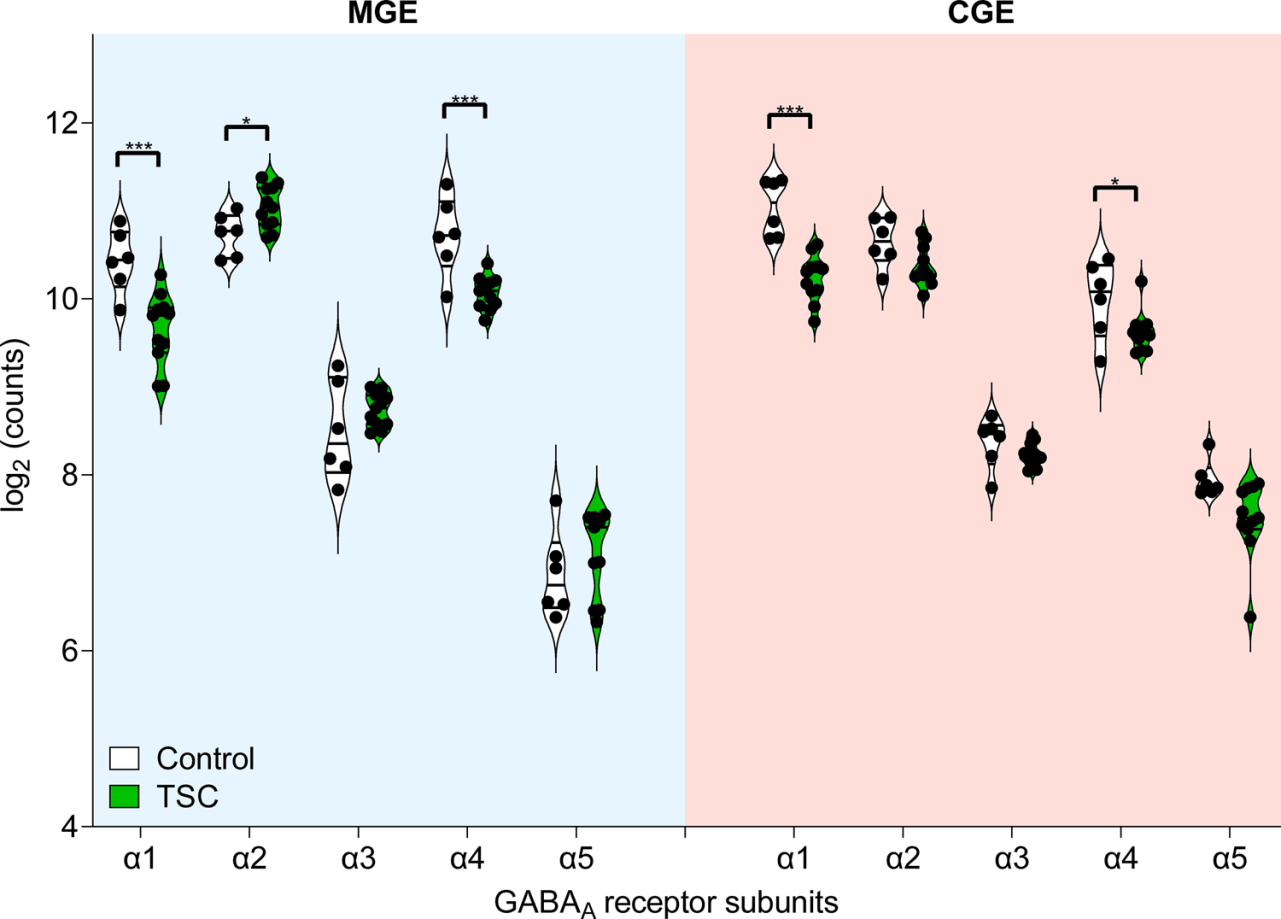
Expression of GABA_A_ receptor subunits in the medial ganglionic eminence (MGE) and caudal ganglionic eminence (CGE) in the tuberous sclerosis complex (TSC). The figure consists of two panels. The left panel represents the expression of GABA_A_ receptor subunits (a1–5) in the MGE, indicated by a blue background. The right panel shows the expression of the same subunits in the CGE, represented by a red background. Each panel displays two sets of violin plots for each subunit, one for control cases depicted in white and the other for TSC cases shown in green. In both the MGE and CGE, there is a significant downregulation of GABA_A_ receptor subunits α1 and α4 in TSC compared to controls. Notably, only in the MGE, there is an upregulation of α2 subunits in TSC cases. Violin plots represent the distribution of expression levels, with wider sections indicating a higher density of expression values. Statistical significance is denoted as * (*p *< 0.05) and *** (*p* < 0.001)

The comparison of control and TSC samples revealed multiple changes in both MGE- and CGE-derived interneurons. However, certain results suggested an immature phenotype in the MGE-derived interneurons. One of the key differences observed was the expression of the GABA_A_ receptor subunit α2. While the expression of this subunit was upregulated in MGE-derived interneurons, it was downregulated in CGE-derived interneurons (Fig. 3). Studies have previously indicated a gradual increase in α1 subunit and decrease in α2/α3 during development [18].

### Parvalbumin and somatostatin specific expression of GABAA receptor subunits

To investigate whether the immature phenotype observed in the medial ganglionic eminence (MGE) is specific to a particular subpopulation of interneurons or if it affects all interneuron subpopulations originating from the MGE, we examined various markers of maturity. Initially, we focused on the GABA_A_ receptor subunits and the *NKCC1/KCC2* ratio. Additionally, we explored other markers including RNA binding fox-1 homolog 3 (*RBFOX3; NeuN*), Microtubule Associated Protein 2 *(MAP2*), tubulin beta 3 class III (*TUBB3*), and stathmin 1 (*STMN1*).

**Fig. 4:**
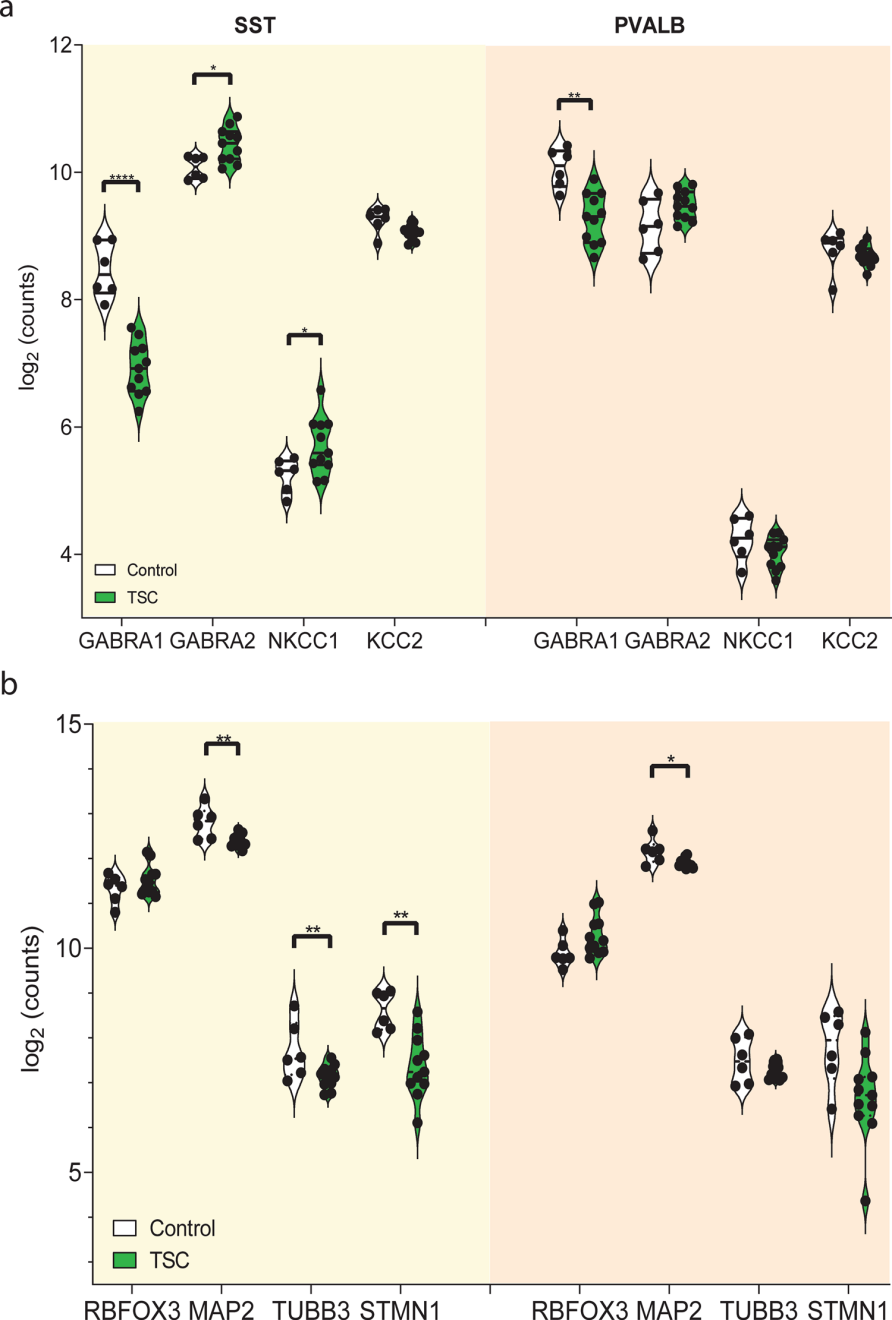
Immaturity phenotype of somatostatin+ (SST) and parvalbumin+ (PVALB) interneurons. The figure comprises two panels. The left panel represents the expression of immaturity markers in SST+ interneurons, depicted by the yellow background. The right panel displays the same markers in PVALB+ interneurons, represented by the orange background. Each panel includes four sets of violin plots, with control cases shown in white and TSC cases depicted in green. **a** In both SST+ and PVALB+ interneurons, there is a significant downregulation of the GABA_A_ receptor subunit α1 (*GABRA1*). However, distinctive differences emerge between the two subpopulations. Specifically, only SST+ interneurons show upregulation of GABA_A_ receptor subunit α2 (*GABRA2*), indicating a differential regulation of GABA receptor expression. Furthermore, SST+ interneurons exhibit upregulation of *NKCC1*, suggesting a more immature phenotype and altered chloride homeostasis compared to PVALB+ interneurons. **b** No significant differences are observed between control and TSC cases for *RBFOX3* (NeuN) expression in both SST+ and PVALB+ interneurons. However, both SST+ and PVALB+ interneurons show downregulation of *MAP2* expression in TSC compared to controls, indicating a common disruption in *MAP2* levels. Remarkably, only SST+ interneurons exhibit downregulation of *TUBB3* and *STMN1*, indicating a more immature phenotype of these interneurons in TSC. Violin plots illustrate the distribution of expression levels, with wide sections representing a higher density of expression values. Statistical significance is denoted as * (*p* < 0.05), ** (*p* < 0.01), and **** (*p* < 0.0001)

First, we examined the expression of GABA_A_ receptor subunits in the MGE-derived interneuron populations. We observed a significant downregulation of the GABA_A_ receptor subunit α1 (*GABRA1*) in both somatostatin (*SST*) and parvalbumin (*PVALB*) interneurons, indicating a shared immaturity phenotype. However, distinctive differences emerged between the two subpopulations. Specifically, only SST interneurons showed upregulation of the GABA_A_ receptor subunit α2 (*GABRA2*), suggesting a differential regulation of GABA receptor expression. Additionally, the α4 subunit was downregulated in both subpopulations (not shown). Next, we investigated the *NKCC1/KCC2* ratio, which is indicative of chloride homeostasis and neuronal maturation. In SST-positive interneurons, we found upregulation of *NKCC1*, suggesting an immature phenotype and altered chloride homeostasis (Fig. 4a).

In contrast, PVALB+ interneurons did not exhibit significant changes in the *NKCC1/KCC2* ratio, suggesting a relatively more mature state compared to SST+ interneurons. To further explore the maturity of MGE-derived interneurons, we examined additional markers. We found no significant differences in *RBFOX3* (*NeuN*) expression between control and TSC cases in both SST+ and PVALB+ interneurons. This suggests that *RBFOX3*, a marker associated with neuronal maturation, is not affected in the context of the immaturity phenotype observed. However, both SST+ and PVALB+ interneurons exhibited downregulation of *MAP2* expression in TSC cases compared to controls, indicating a common disruption in *MAP2* levels associated with the immaturity phenotype. Notably, only SST+ interneurons showed downregulation of *TUBB3* and *STMN1*, indicating a more immature phenotype specifically in these interneurons in TSC (Fig. 4b). These findings provide evidence that SST+ interneurons exhibit specific molecular markers associated with immaturity, potentially influencing their function properties and connectivity within neural networks.

### Localization of SST + interneurons in the cortex of TSC patients

**Fig. 5:**
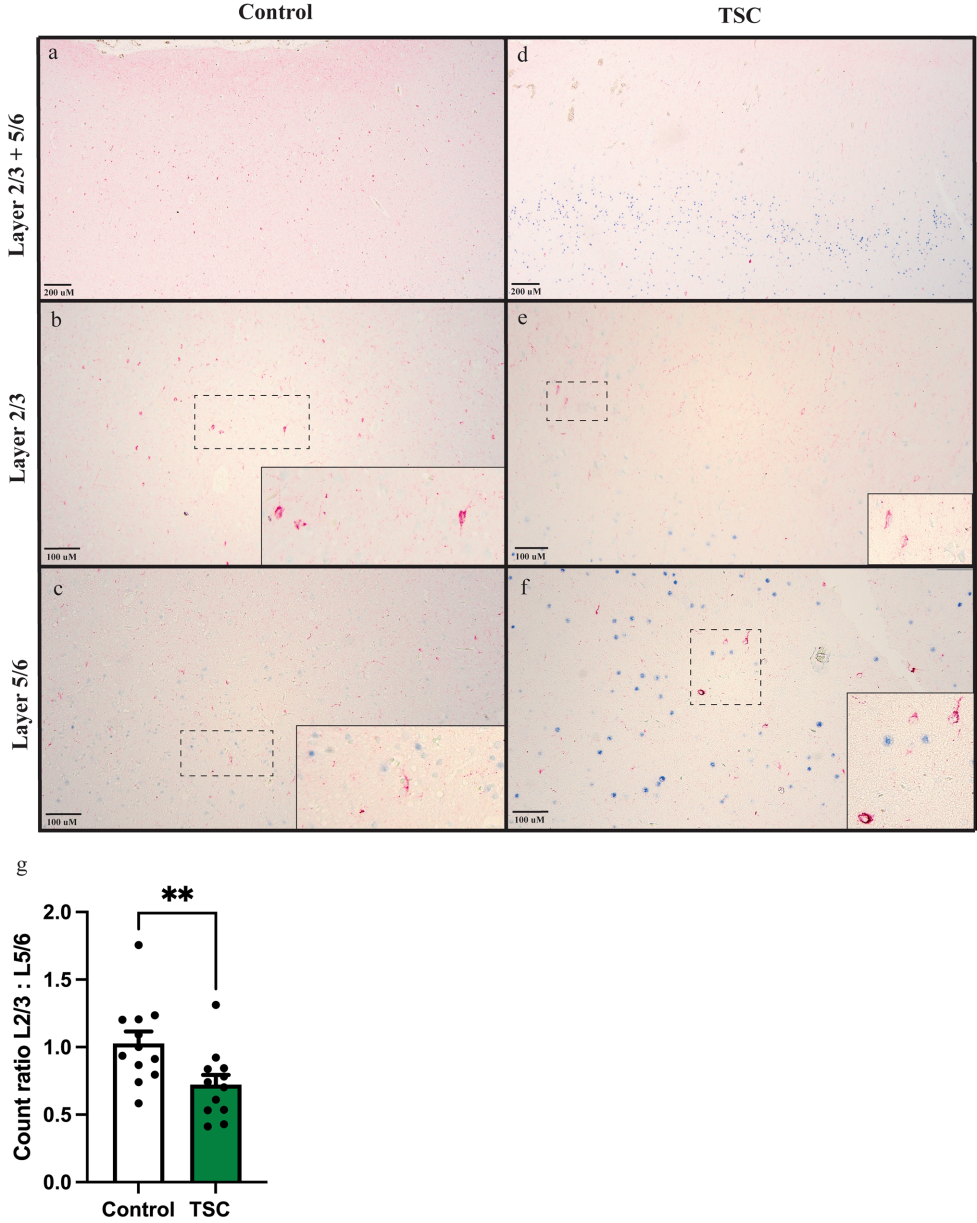
Mislocalization of SST + interneurons in the cortex of individuals with TSC. **a** Representative image of SST (red) and FOXP2 (blue) immunohistochemical staining in the control case. FOXP2 serves as a reference marker for Layer 5/6 of the cortex. **b** Representative image of L2/3 in which SST+ cells were counted in control, including an inset showing a higher magnification image of SST+ neurons in this area. **c** Representative image of L5/6 in which SST+ cells were counted in control, including an inset showing a higher magnification image of SST+ neurons in this area. **d** Immunohistochemical staining of SST (red) and FOXP2 (blue) in a TSC case, highlighting alterations in the distribution and expression patterns compared to the control case. **e** Representative image of L2/3 in which SST+ cells were counted in TSC, including an inset showing a higher magnification image of SST+ neurons in this area. **f** Representative image of L5/6 in which SST+ cells were counted in TSC, including an inset showing a higher magnification image of SST+ neurons in this area. **g** Quantification of the ratio between SST+ interneuron cell counts in layer 2/3 and layer 5/6. A Mann–Whitney *U* test revealed a significant difference between the control and TSC cases (** *p* < 0.01). Error bars represent the standard error of the mean (SEM)

The layer specific spatial distribution of GABAergic interneurons in the cortex contributes to the formation of local neuronal networks and the connections formed among different neuronal subtypes. As shown previously, SST+ interneurons show a more immature phenotype compared to other interneuron subpopulations. Therefore, to explore the correlation between immaturity and the positioning of SST+ interneurons in the cortex of individuals with TSC, we conducted immunohistochemical double-labeling staining. SST+ interneurons are localized in superficial (L2/3) layers and deep (L5/6) cortical layers [34, 36, 63]. To identify L5/6, antibody FOXP2 was used as a reference [16, 31]. Frontal cortex from both control (*n* = 3) and TSC (*n* = 3) was used in these experiments and direct comparisons were made. For each sample, four different areas were imaged, and the cell count of SST+ cells was determined in both L2/3 and L5/6 respectively (Fig. 5a, b). A ratio between the cell counts in these layers was calculated to determine the difference in localization of SST+ interneurons in control and TSC tissue. When comparing these ratios between control and TSC, we observed a significantly lower L2/3-L5/6 ratio in TSC. This indicates a significant reduction in the number of SST+ interneurons in the L2/3 region in individuals with TSC compared to the number of SST+ interneurons in L5/6 (Fig. 5c), suggesting a possible migration problem of these interneurons.

### Functional assessment of GABAAR subunit changes and proposed immaturity in *Xenopus* oocytes

Considering our earlier finding of altered GABA_A_ receptor expression, we sought to investigate the affinity of GABA_A_ receptors using TSC and control membranes injected into *Xenopus* oocytes.

Although the oocyte system is not SST-specific, its exploration provides valuable insights into how disruptions in SST+ interneurons could reverberate across neural networks, even when accounting for the diversity of other cell types. Our analysis revealed no significant differences in GABA_A_ receptor affinity between the TSC and control groups (Supplementary Fig. 1). However, it is worth noting that this affinity measurement encompassed all interneuron subpopulations, potentially negating any specific effect of the SST+ interneuron population. Given the implication of SST+ interneurons in network dysfunction and epilepsy, it is plausible that their distinct characteristic may contribute to differential effects on GABA_A_ receptor affinity.

To gain further insight into the possible functional immaturity of interneurons and the consequences of this, we also examined the GABA reversal potential (E_GABA_) in the oocyte system. Interestingly, we found a more depolarized E_GABA_ in TSC compared to the values that have previously been reported in controls and/or in other pathological cortical samples [46, 53], indicating alterations in inhibitory neurotransmission (Supplementary Fig. 2). The observed changes in E_GABA_ provide additional evidence supporting the notion of immaturity in the GABAergic neuron populations. Specifically, the shift towards a more depolarized E_GABA_ is in line with an imbalance in chloride transporters, potentially reflecting an immature state characterized by higher expression of *NKCC1* and lower expression of *KCC2*. These findings highlight the developmental aspect of interneurons and their potential contribution to the overall network excitability and functional maturation, confirming previous studies [53].

**Fig. 6:**
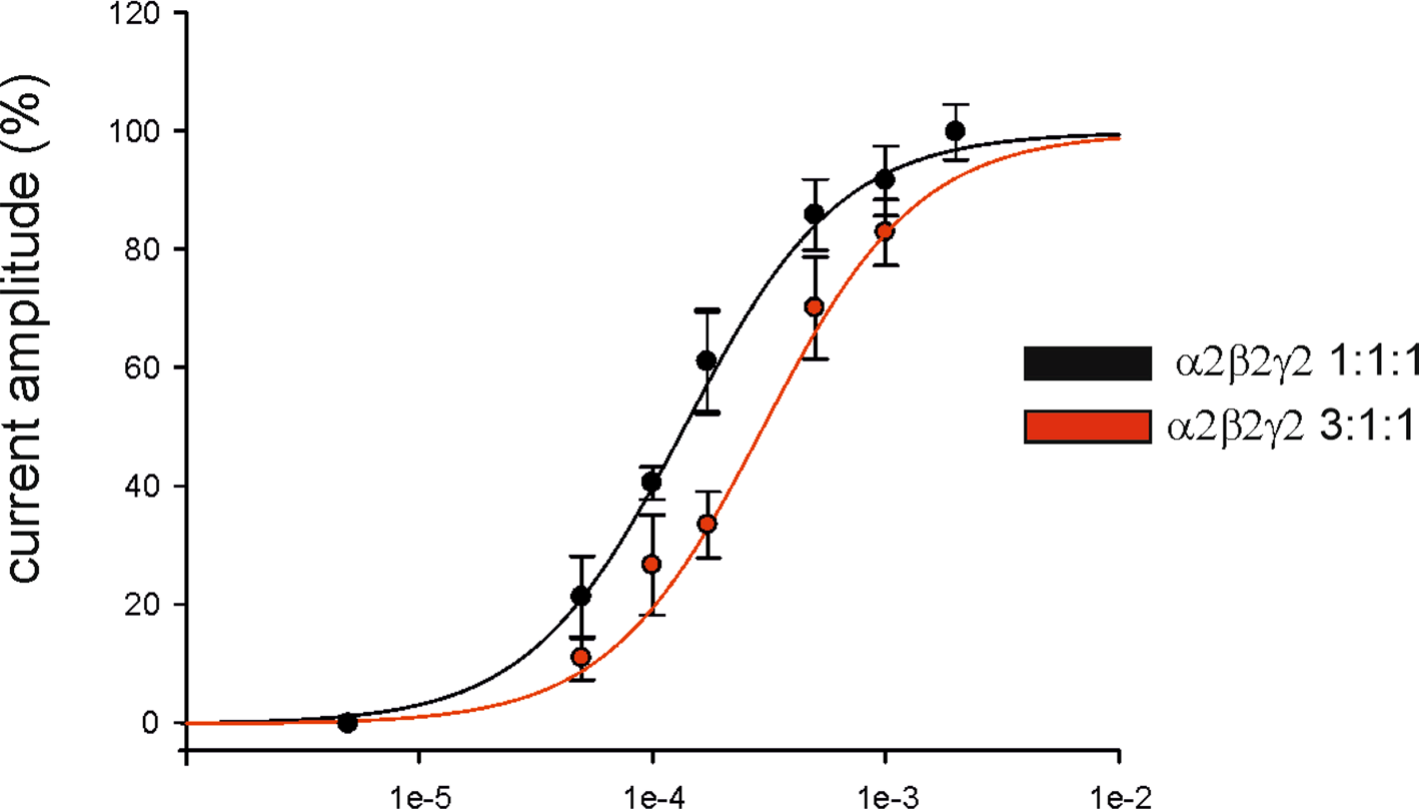
GABA_A_R apparent affinity in oocytes injected with GABA_A_ receptor subunits. The graph shows the amplitudes (as mean ± standard error of the mean [SEM]) of currents evoked at different GABA concentrations, expressed as a percentage of the maximal current evoked (● = 1265 ± 480 nA; ● = 236 ± 176 nA) and best fitted by Hill curves. The EC_50_ values and n_H_ were 137 ± 5.5 μM and 1.29 ± 0.5 in oocytes injected with α2β2γ2 (1:1:1 ratio, *n* = 14) and 298 ± 6 μM and 1.3 ± 0.02 (*n* = 10) in oocytes injected with the upregulated isoform (α2β2γ2, 3:1:1 ratio). Note the rightward shift of the α2β2γ2 3:1:1 receptors

Next, we aimed to further investigate the functional consequences of (GABRA2) GABA_A_ receptor subunit α2 upregulation. In our previous data analysis, we determined that *GABRA2* upregulation was found in the immature population of SST+ interneurons. Therefore, to explore this, we conducted additional experiments involving injections in *Xenopus* oocytes, allowing us to assess the overall effect on receptor affinity. The upregulation of α2 was simulated by increasing the ratio of α2 compared to the other subunits (β2 and γ2). We compared two different ratios of GABA_A_ receptor subunits (α2β2γ2, 1:1:1 and α2β2γ2, 3:1:1). The results demonstrated that the alteration of subunit ratios has a notable effect on the GABA_A_ receptor affinity. Oocytes injected with the 3:1:1 ratio exhibited a significantly lower receptor affinity for GABA compared to those injected with the 1:1:1 ratio (Fig. 6), which was in line with previous findings [26]. This suggests that the upregulation of GABA_A_ receptor subunit α2 subunits in overall receptor composition leads to an overall reduction in receptor affinity. Moreover, this suggests that GABA_A_ receptor subunit α2 upregulation potentially may impact the inhibitory neurotransmission within the SST + population.

## Discussion

GABAergic interneurons play a crucial role in the functioning of neural circuits within the brain [60]. As local neurons, they precisely modulate the excitatory output of principal neurons, contributing to a balanced excitation–inhibition ratio, synchronization of neuronal firing, and the shaping of the receptive field. This precise regulation influences information processing and cognitive development [64]. In the context of tuberous sclerosis complex (TSC), the dysfunction of interneurons has been implicated in the pathophysiology of the disorder. TSC is characterized by abnormal cellular growth and the formation of cortical tubers, which disrupt the architecture and function of affected brain regions. Disrupted interneuron function can lead to an imbalance between excitation and inhibition, resulting in abnormal neuronal network activity and the manifestation of TSC-related neurological symptoms [27].

In this study, we focused on investigating the specific GABAergic subpopulations within TSC to gain insights into their role in the aberrant network activity observed in the disorder. Examining the expression patterns of different interneuron subpopulations, we aimed to shed light on their contributions to the immature and dysfunctional neural circuits observed in TSC. Our investigation particularly centered around the MGE-derived and CGE-derived interneurons, which are known to exhibit diverse molecular profiles and functional properties [56]. By analyzing snRNA-seq data, we were able to distinguish these interneuron subpopulations and further explore their characteristics separately. Within the MGE-derived interneurons, our findings revealed that the SST+ interneuron population exhibited an immature phenotype. Other subpopulations showed less changes, possibly due to a difference in number of neurons between control and TSC. However, this observation aligns with previous studies in TSC and focal cortical dysplasia (FCD), suggesting that SST+ interneurons may undergo disrupted maturation and fail to properly switch from excitatory to inhibitory neurotransmission during development [68]. This immaturity could contribute to the dysfunctional network activity and altered neuronal excitability observed in TSC.

Furthermore, the downregulation of *GABRA1* and upregulation of *GABRA2*, key subunits of the GABA_A_ receptor, in SST+ interneurons present an intriguing finding. Given that these interneurons exhibit a more immature and potentially reduced inhibitory phenotype in TSC, it is reasonable to consider the implications of altered GABAergic signaling in these cells. GABA_A_ receptors are crucial for mediating inhibitory neurotransmission in the brain, and their subunit composition plays a pivotal role in determining receptor properties and functional output [19, 55]. The downregulation of *GABRA1* and upregulation of *GABRA2* in SST+ interneurons suggest a potential shift in the composition and function of GABA_A_ receptors within this specific population. This alteration may have significant consequences for interneuronal network dynamics and the overall balance of excitation and inhibition in TSC. Our findings of a reduced affinity for GABA in *Xenopus* oocytes injected with higher ratios of α2 human cDNA support this hypothesis. Notably, when we transplant all the GABA_A_ receptors from TSC and control tissues, we did not observe differences in the apparent receptor affinity. This evidence suggests the presence of a GABAergic impairment affecting a specific subtype of GABAA receptor. Other electrophysiological recordings (such as patch-clamp on specific interneuronal populations) may provide further details on the effect of GABA_A_R subunit dysregulation in SST+ interneurons. However, these methods pose technical challenges and are constrained by the availability of fresh human TSC tissue. Furthermore, the changes in GABA_A_ receptor subunits in SST+ interneurons could be seen as analogous to a similar alteration occurring in excitatory neurons. If these SST+ interneurons exhibit a reduced inhibitory tone, which could be the result of an increase in both glutamate and GABA release or co-release, the downregulation of specific GABA_A_ receptor subunits could be considered an adaptive response to maintain appropriate signaling within the network. One possible explanation for the downregulation of *GABRA1* and upregulation of *GABRA2* in SST+ interneurons is the presence of compensatory mechanisms aimed at maintaining balanced network activity.

Moreover, the identification of a change in the *NKCC1/KCC2* ratio exclusively in SST+ interneurons is in line with the prior findings highlighting an immature phenotype exhibited by SST+ interneurons again leading to a reduced inhibitory tone [58]. Additionally, the observed changes in E_GABA_, reflecting a shift toward a more depolarized state, provides additional evidence supporting the notion of immaturity in these interneurons. This alteration is consistent with an imbalance in chloride transporters, potentially indicating a developmental state marked by higher *NKCC1* expression and lower or stable *KCC2* expression [6, 25]. However, it is important to acknowledge that the absence of detectable change in the *NKCC1/KCC2* ratio in other interneuronal subpopulations does not rule out differences in chloride transporter expression or function. This discrepancy could arise from nuanced variations at the protein level, including post-translational modifications or differences in membrane localization.

Another important aspect of SST+ interneurons is their ability to exhibit diverse responses to incoming activity underscoring their unique projections through multiple layers and specific interactions with dendrites. In addition to receiving inputs from GABAergic interneurons, such as VIP+ interneurons, and excitatory projection neurons [65], SST+ interneurons intricately process information from varied sources, thereby enabling precise modulation of network activity. The inhibitory connections formed by SST+ interneurons serve to regulate the excitability of the network. By receiving inputs from other GABAergic interneurons, SST+ interneurons can effectively control the activity of excitatory neurons. Through the release of the inhibitory neurotransmitter GABA, SST+ interneurons can dampen excessive excitatory signaling, preventing runaway excitation and maintaining network stability [52]. At the same time, SST+ interneurons also establish connections with excitatory projection neurons. This connectivity allows SST+ interneurons to influence the activity of excitatory neurons and shape their firing patterns. By modulating the timing and strength of excitatory inputs onto target neurons, somatostatin interneurons can fine-tune the network's information processing capabilities [32, 61].

Furthermore, we explored the localization of SST+ interneurons within TSC brain tissue. Here we found that SST+ interneurons in TSC are mainly located in deeper cortical layers L5/6, while control tissue had SST+ interneurons evenly spread in superficial layers L2/3 as well as L5/6. This indicates a difference in migration patterns of these SST+ interneurons in TSC. One key feature of TSC, however, is the cortical dyslamination that occurs during development [11]. This results in improper formation of the cortical layers, which in turn potentially impacts the localization of certain interneuron subpopulations.

In the domain of epilepsy research, the intricate network of cortical GABAergic interneurons has become an area of interest, providing a nuanced comprehension of their varied roles in shaping seizures. Recent studies have revealed that alterations or immaturity in GABAergic functioning, particularly involving SST+ interneurons, are associated with seizure disorders. Notably, SST+ interneurons in specific regions of the brain exhibit increased axonal sprouting in an epileptogenesis model [8, 47, 67], as well as heightened excitatory input [20], potentially influencing their ability to synchronize network inhibition. The intricate connection between SST+ interneurons and seizures goes beyond a simple association, exemplified by a decrease in SST+ interneuron activity in the neocortex observed in Dravet syndrome, an example of a monogenic epilepsy caused by a pathogenic variant in the *SCN1A* gene [57]. These findings highlight the complexity of GABAergic interneuron involvement in epilepsy, suggesting that targeted interventions to modulate specific interneuron subpopulations, such as SST+ interneurons, may hold therapeutic advantages in managing seizures. Furthermore, the proposition of utilizing somatostatin neuropeptide receptors as a potential avenue for anticonvulsant therapies adds a layer of promise to precision medicine in epilepsy management [13]. This evolving understanding of the intricate roles played by different GABAergic subpopulations not only refines therapeutic considerations for seizure control but also opens new possibilities for addressing the broader spectrum of neuropsychiatric manifestations associated with the tuberous sclerosis complex (TAND) [9, 10, 49]. Embracing the heterogeneity among GABAergic interneurons paves the way for precision medicine applications, offering tailored therapeutic strategies for individuals with TSC and associated neuropsychiatric disorders.

Future research should focus on elucidating the functional properties of SST+ interneurons in TSC. Understanding the specific roles and contributions of these interneurons to the aberrant network activity observed in TSC will provide valuable insights into the pathophysiology of the disorder. Several avenues of investigation can be pursued in this regard. Firstly, it is crucial to investigate the electrophysiological properties of SST+ interneurons in TSC. Characterizing their firing patterns, membrane properties, and synaptic connectivity will provide a deeper understanding of how they functionally integrate within the neuronal circuits. Electrophysiological recordings, such as whole-cell patch-clamp techniques, can be employed to examine the excitatory and inhibitory synaptic inputs, action potential firing patterns, and intrinsic properties of these interneurons. Comparative studies between SST+ interneurons from TSC samples and healthy control tissue will help identify specific alterations in their functional properties. Furthermore, studying the synaptic connections of SST+ interneurons will provide insights into their circuit-level interactions and potential dysfunctions in TSC. This can be achieved through techniques like optogenetics and calcium imaging, which allow for the manipulation and monitoring of neuronal activity in a precise and cell type-specific manner. Investigating the connectivity patterns and synaptic dynamics involving SST+ interneurons will help unravel the disrupted inhibitory-excitatory balance and aberrant network activity associated with TSC. Finally, functional studies using human-derived models in vitro should also consider the interaction of SST + interneurons with other interneuron subtypes and excitatory neurons within the TSC brain. Investigating the synaptic connections, activity patterns, and plasticity mechanisms involved in these interactions will provide a comprehensive understanding of the disrupted neuronal networks in TSC.

In conclusion, our study provides evidence that SST + interneurons represent a distinct and immature interneuron sub-population in TSC. The immaturity of these interneurons, coupled with their altered functional properties, may contribute to the disrupted network activity and dysfunctional neural circuits observed in TSC. Future research should focus on elucidating the precise functional roles of somatostatin interneurons and their interactions within the TSC brain to further unravel the underlying mechanisms of the disorder and identify potential therapeutic targets.

### Supplementary Information

Below is the link to the electronic supplementary material.

Supplementary file1 (DOCX 203 KB)

## Data Availability

The data analyzed in this study was obtained from Biotech Research & Innovation Center (BRIC), University of Copenhagen, Denmark, the following licenses/restrictions apply: the third-party producer of the data will not allow the data to be released to the public domain until September 2024. Requests to access these datasets should be directed to e.aronica@amsterdamumc.nl.

## References

[1] Alfano V, Romagnolo A, Mills JD, Cifelli P, Gaeta A, Morano A, Mühlebner A, Aronica E, Palma E, Ruffolo G (2022). Unexpected effect of IL-1β on the function of GABAA receptors in pediatric focal cortical dysplasia. Brain Sci.

[2] Aronica E, Boer K, Redeker S, Spliet WGM, van Rijen PC, Troost D, Gorter JA (2007). Differential expression patterns of chloride transporters, Na+-K+-2Cl–cotransporter and K+-Cl–cotransporter, in epilepsy-associated malformations of cortical development. Neuroscience.

[3] Bandler RC, Mayer C, Fishell G (2017). Cortical interneuron specification: the juncture of genes, time and geometry. Curr Opin Neurobiol.

[4] Bassetti D, Luhmann HJ, Kirischuk S (2021). Effects of mutations in TSC genes on neurodevelopment and synaptic transmission. Int J Mol Sci.

[5] Batiuk MY, Tyler T, Dragicevic K, Mei S, Rydbirk R, Petukhov V, Deviatiiarov R, Sedmak D, Frank E, Feher V, Habek N, Hu Q, Igolkina A, Roszik L, Pfisterer U, Garcia-Gonzalez D, Petanjek Z, Adorjan I, Kharchenko PV, Khodosevich K (2022). Upper cortical layer–driven network impairment in schizophrenia. Sci Adv.

[6] Ben-Ari Y, Khalilov I, Kahle KT, Cherubini E (2012). The GABA excitatory/inhibitory shift in brain maturation and neurological disorders. Neurosci Rev J Bringing Neurobiol Neurol Psychiatry.

[7] Brooks-Kayal AR, Shumate MD, Jin H, Rikhter TY, Coulter DA (1998). Selective changes in single cell GABAA receptor subunit expression and function in temporal lobe epilepsy. Nat Med.

[8] Buckmaster PS, Wen X (2011). Rapamycin suppresses axon sprouting by somatostatin interneurons in a mouse model of temporal lobe epilepsy. Epilepsia.

[9] Cellot G, Cherubini E (2014). GABAergic signaling as therapeutic target for autism spectrum disorders. Front Pediatr.

[10] Cherubini E, Di Cristo G, Avoli M (2021). Dysregulation of GABAergic signaling in neurodevelomental disorders: targeting cation-chloride co-transporters to re-establish a proper E/I balance. Front Cell Neurosci.

[11] Curatolo P, Specchio N, Aronica E (2022). Advances in the genetics and neuropathology of tuberous sclerosis complex: edging closer to targeted therapy. Lancet Neurol.

[12] de Vries PJ, Belousova E, Benedik MP, Carter T, Cottin V, Curatolo P, D’Amato L, Beure d’Augères G, Ferreira JC, Feucht M, Fladrowski C, Hertzberg C, Jozwiak S, Lawson JA, Macaya A, Marques R, Nabbout R, O’Callaghan F, Qin J, Sander V, Sauter M, Shah S, Takahashi Y, Touraine R, Youroukos S, Zonnenberg B, Kingswood JC, Jansen AC, TOSCA Consortium and TOSCA Investigators (2020) Natural clusters of tuberous sclerosis complex (TSC)-associated neuropsychiatric disorders (TAND): new findings from the TOSCA TAND research project. J Neurodev Disord 12:24. doi: 10.1186/s11689-020-09327-0 10.1186/s11689-020-09327-0 PMC7465404 32873244

[13] Dobolyi A, Kékesi KA, Juhász G, Székely AD, Lovas G, Kovács Z (2014). Receptors of peptides as therapeutic targets in epilepsy research. Curr Med Chem.

[14] Fatemi SH, Halt AR, Stary JM, Kanodia R, Schulz SC, Realmuto GR (2002). Glutamic acid decarboxylase 65 and 67 kDa proteins are reduced in autistic parietal and cerebellar cortices. Biol Psychiatry.

[15] Fatemi SH, Reutiman TJ, Folsom TD, Thuras PD (2009). GABAA receptor downregulation in brains of subjects with autism. J Autism Dev Disord.

[16] Ferland RJ, Cherry TJ, Preware PO, Morrisey EE, Walsh CA (2003). Characterization of Foxp2 and Foxp1 mRNA and protein in the developing and mature brain. J Comp Neurol.

[17] Fishell G, Kepecs A (2020). Interneuron types as attractors and controllers. Annu Rev Neurosci.

[18] Galanopoulou AS (2008). GABAA receptors in normal development and seizures: friends or foes?. Curr Neuropharmacol.

[19] Ghit A, Assal D, Al-Shami AS, Hussein DEE (2021). GABAA receptors: structure, function, pharmacology, and related disorders. J Genet Eng Biotechnol.

[20] Halabisky B, Parada I, Buckmaster PS, Prince DA (2010). Excitatory input onto hilar somatostatin interneurons is increased in a chronic model of epilepsy. J Neurophysiol.

[21] Hao Y, Hao S, Andersen-Nissen E, Mauck WM, Zheng S, Butler A, Lee MJ, Wilk AJ, Darby C, Zager M, Hoffman P, Stoeckius M, Papalexi E, Mimitou EP, Jain J, Srivastava A, Stuart T, Fleming LM, Yeung B, Rogers AJ, McElrath JM, Blish CA, Gottardo R, Smibert P, Satija R (2021). Integrated analysis of multimodal single-cell data. Cell.

[22] Hashimoto T, Nguyen QL, Rotaru D, Keenan T, Arion D, Beneyto M, Gonzalez-Burgos G, Lewis DA (2009). Protracted developmental trajectories of GABAA receptor α1 and α2 subunit expression in primate prefrontal cortex. Biol Psychiatry.

[23] Hodges SL, Lugo JN (2020). Therapeutic role of targeting mTOR signaling and neuroinflammation in epilepsy. Epilepsy Res.

[24] van Hugte EJH, Schubert D, Nadif Kasri N (2023). Excitatory/inhibitory balance in epilepsies and neurodevelopmental disorders: depolarizing γ-aminobutyric acid as a common mechanism. Epilepsia.

[25] Kaila K, Price TJ, Payne JA, Puskarjov M, Voipio J (2014). Cation-chloride cotransporters in neuronal development, plasticity and disease. Nat Rev Neurosci.

[26] Karim N, Wellendorph P, Absalom N, Johnston GAR, Hanrahan JR, Chebib M (2013). Potency of GABA at human recombinant GABAA receptors expressed in Xenopus oocytes: a mini review. Amino Acids.

[27] Katsarou A-M, Moshé SL, Galanopoulou AS (2017). Interneuronopathies and their role in early life epilepsies and neurodevelopmental disorders. Epilepsia Open.

[28] Kepecs A, Fishell G (2014). Interneuron cell types are fit to function. Nature.

[29] Kessaris N, Magno L, Rubin AN, Oliveira MG (2014). Genetic programs controlling cortical interneuron fate. Curr Opin Neurobiol.

[30] Krishnaswami SR, Grindberg RV, Novotny M, Venepally P, Lacar B, Bhutani K, Linker SB, Pham S, Erwin JA, Miller JA, Hodge R, McCarthy JK, Kelder M, McCorrison J, Aevermann BD, Fuertes FD, Scheuermann RH, Lee J, Lein ES, Schork N, McConnell MJ, Gage FH (2016). Lasken RS (2016) Using single nuclei for RNA-seq to capture the transcriptome of postmortem neurons. Nat Protoc.

[31] Lai CSL, Gerrelli D, Monaco AP, Fisher SE, Copp AJ (2003). FOXP2 expression during brain development coincides with adult sites of pathology in a severe speech and language disorder. Brain J Neurol.

[32] Liguz-Lecznar M, Urban-Ciecko J, Kossut M (2016). Somatostatin and somatostatin-containing neurons in shaping neuronal activity and plasticity. Front Neural Circuits.

[33] Love MI, Huber W, Anders S (2014). Moderated estimation of fold change and dispersion for RNA-seq data with DESeq2. Genome Biol.

[34] Ma Y, Hu H, Berrebi AS, Mathers PH, Agmon A (2006). Distinct subtypes of somatostatin-containing neocortical interneurons revealed in transgenic mice. J Neurosci.

[35] Malik R, Pai EL-L, Rubin AN, Stafford AM, Angara K, Minasi P, Rubenstein JL, Sohal VS, Vogt D (2019). Tsc1 represses parvalbumin expression and fast-spiking properties in somatostatin lineage cortical interneurons. Nat Commun.

[36] Markram H, Toledo-Rodriguez M, Wang Y, Gupta A, Silberberg G, Wu C (2004). Interneurons of the neocortical inhibitory system. Nat Rev Neurosci.

[37] Marín O, Rubenstein JL (2001). A long, remarkable journey: tangential migration in the telencephalon. Nat Rev Neurosci.

[38] Mi D, Li Z, Lim L, Li M, Moissidis M, Yang Y, Gao T, Hu TX, Pratt T, Price DJ, Sestan N, Marín O (2018). Early emergence of cortical interneuron diversity in the mouse embryo. Science.

[39] Mueller-Buehl C, Wegrzyn D, Bauch J, Faissner A (2023). Regulation of the E/I-balance by the neural matrisome. Front Mol Neurosci.

[40] Mühlebner A, Bongaarts A, Sarnat HB, Scholl T, Aronica E (2019). New insights into a spectrum of developmental malformations related to mTOR dysregulations: challenges and perspectives. J Anat.

[41] Najm I, Lal D, Alonso Vanegas M, Cendes F, Lopes-Cendes I, Palmini A, Paglioli E, Sarnat HB, Walsh CA, Wiebe S, Aronica E, Baulac S, Coras R, Kobow K, Cross JH, Garbelli R, Holthausen H, Rössler K, Thom M, El-Osta A, Lee JH, Miyata H, Guerrini R, Piao Y-S, Zhou D, Blümcke I (2022). The ILAE consensus classification of focal cortical dysplasia: an update proposed by an ad hoc task force of the ILAE diagnostic methods commission. Epilepsia.

[42] Nery S, Fishell G, Corbin JG (2002). The caudal ganglionic eminence is a source of distinct cortical and subcortical cell populations. Nat Neurosci.

[43] Northrup H, Aronow ME, Bebin EM, Bissler J, Darling TN, de Vries PJ, Frost MD, Fuchs Z, Gosnell ES, Gupta N, Jansen AC, Jóźwiak S, Kingswood JC, Knilans TK, McCormack FX, Pounders A, Roberds SL, Rodriguez-Buritica DF, Roth J, Sampson JR, Sparagana S, Thiele EA, Weiner HL, Wheless JW, Towbin AJ, Krueger DA, International Tuberous Sclerosis Complex Consensus Group (2021) Updated International Tuberous Sclerosis Complex Diagnostic Criteria and Surveillance and Management Recommendations. Pediatr Neurol 123:50–66. doi: 10.1016/j.pediatrneurol.2021.07.011 10.1016/j.pediatrneurol.2021.07.011 34399110

[44] Palma E, Esposito V, Mileo AM, Di Gennaro G, Quarato P, Giangaspero F, Scoppetta C, Onorati P, Trettel F, Miledi R, Eusebi F (2002). Expression of human epileptic temporal lobe neurotransmitter receptors in Xenopus oocytes: an innovative approach to study epilepsy. Proc Natl Acad Sci U S A.

[45] Palma E, Trettel F, Fucile S, Renzi M, Miledi R, Eusebi F (2003). Microtransplantation of membranes from cultured cells to Xenopus oocytes: a method to study neurotransmitter receptors embedded in native lipids. Proc Natl Acad Sci U S A.

[46] Palma E, Amici M, Sobrero F, Spinelli G, Di Angelantonio S, Ragozzino D, Mascia A, Scoppetta C, Esposito V, Miledi R, Eusebi F (2006). Anomalous levels of Cl− transporters in the hippocampal subiculum from temporal lobe epilepsy patients make GABA excitatory. Proc Natl Acad Sci U S A.

[47] Peng Z, Zhang N, Wei W, Huang CS, Cetina Y, Otis TS, Houser CR (2013). A reorganized GABAergic circuit in a model of epilepsy: evidence from optogenetic labeling and stimulation of somatostatin interneurons. J Neurosci.

[48] Pfisterer U, Petukhov V, Demharter S, Meichsner J, Thompson JJ, Batiuk MY, Martinez AA, Vasistha NA, Thakur A, Mikkelsen J, Adorjan I, Pinborg LH, Pers TH, von Engelhardt J, Kharchenko PV (2020). Khodosevich K (2020) Identification of epilepsy-associated neuronal subtypes and gene expression underlying epileptogenesis. Nat Commun.

[49] Pizzarelli R, Cherubini E (2011). Alterations of GABAergic signaling in autism spectrum disorders. Neural Plast.

[50] Powell EM (2013). Interneuron development and epilepsy: early genetic defects cause long-term consequences in seizures and susceptibility. Epilepsy Curr.

[51] Raol YH, Lund IV, Bandyopadhyay S, Zhang G, Roberts DS, Wolfe JH, Russek SJ, Brooks-Kayal AR (2006). Enhancing GABAA receptor α1 subunit levels in hippocampal dentate gyrus inhibits epilepsy development in an animal model of temporal lobe epilepsy. J Neurosci.

[52] Rudy B, Fishell G, Lee S, Hjerling-Leffler J (2011). Three groups of interneurons account for nearly 100% of neocortical GABAergic neurons. Dev Neurobiol.

[53] Ruffolo G, Iyer A, Cifelli P, Roseti C, Mühlebner A, van Scheppingen J, Scholl T, Hainfellner JA, Feucht M, Krsek P, Zamecnik J, Jansen FE, Spliet WGM, Limatola C, Aronica E, Palma E (2016). Functional aspects of early brain development are preserved in tuberous sclerosis complex (TSC) epileptogenic lesions. Neurobiol Dis.

[54] Ruffolo G, Di Bonaventura C, Cifelli P, Roseti C, Fattouch J, Morano A, Limatola C, Aronica E, Palma E, Giallonardo AT (2018). A novel action of lacosamide on GABAA currents sets the ground for a synergic interaction with levetiracetam in treatment of epilepsy. Neurobiol Dis.

[55] Sallard E, Letourneur D, Legendre P (2021). Electrophysiology of ionotropic GABA receptors. Cell Mol Life Sci.

[56] Sultan K, Brown K, Shi S-H (2013). Production and organization of neocortical interneurons. Front Cell Neurosci.

[57] Tai C, Abe Y, Westenbroek RE, Scheuer T, Catterall WA (2014). Impaired excitability of somatostatin- and parvalbumin-expressing cortical interneurons in a mouse model of Dravet syndrome. Proc Natl Acad Sci U S A.

[58] Talos DM, Sun H, Kosaras B, Joseph A, Folkerth RD, Poduri A, Madsen JR, Black PM, Jensen FE (2012). Altered inhibition in tuberous sclerosis and type IIb cortical dysplasia. Ann Neurol.

[59] Talos DM, Jacobs LM, Gourmaud S, Coto CA, Sun H, Lim K-C, Lucas TH, Davis KA, Martinez-Lage M, Jensen FE (2018). Mechanistic target of rapamycin complex 1 and 2 in human temporal lobe epilepsy. Ann Neurol.

[60] Tremblay R, Lee S, Rudy B (2016). GABAergic interneurons in the neocortex: from cellular properties to circuits. Neuron.

[61] Tuncdemir SN, Wamsley B, Stam FJ, Osakada F, Goulding M, Callaway EM, Rudy B, Fishell G (2016). Early somatostatin interneuron connectivity mediates the maturation of deep layer cortical circuits. Neuron.

[62] Vanclooster S, Bissell S, van Eeghen AM, Chambers N, De Waele L, Byars AW, Capal JK, Cukier S, Davis P, Flinn J, Gardner-Lubbe S, Gipson T, Heunis T-M, Hook D, Kingswood JC, Krueger DA, Kumm AJ, Sahin M, Schoeters E, Smith C, Srivastava S, Takei M, Waltereit R, Jansen AC, de Vries PJ (2022). The research landscape of tuberous sclerosis complex-associated neuropsychiatric disorders (TAND)-a comprehensive scoping review. J Neurodev Disord.

[63] Wang Y, Toledo-Rodriguez M, Gupta A, Wu C, Silberberg G, Luo J, Markram H (2004). Anatomical, physiological and molecular properties of Martinotti cells in the somatosensory cortex of the juvenile rat. J Physiol.

[64] Warm D, Schroer J, Sinning A (2022). Gabaergic interneurons in early brain development: conducting and orchestrated by cortical network activity. Front Mol Neurosci.

[65] Wu SJ, Sevier E, Dwivedi D, Saldi G-A, Hairston A, Yu S, Abbott L, Choi DH, Sherer M, Qiu Y, Shinde A, Lenahan M, Rizzo D, Xu Q, Barrera I, Kumar V, Marrero G, Prönneke A, Huang S, Kullander K, Stafford DA, Macosko E, Chen F, Rudy B, Fishell G (2023). Cortical somatostatin interneuron subtypes form cell-type-specific circuits. Neuron.

[66] Yu Y, Zeng Z, Xie D, Chen R, Sha Y, Huang S, Cai W, Chen W, Li W, Ke R, Sun T (2021). Interneuron origin and molecular diversity in the human fetal brain. Nat Neurosci.

[67] Zhang W, Yamawaki R, Wen X, Uhl J, Diaz J, Prince DA, Buckmaster PS (2009). Surviving hilar somatostatin interneurons enlarge, sprout axons, and form new synapses with granule cells in a mouse model of temporal lobe epilepsy. J Neurosci.

[68] Zheng Y, Xu C, Sun J, Ming W, Dai S, Shao Y, Qiu X, Li M, Shen C, Xu J, Fei F, Fang J, Jiang X, Zheng G, Hu W, Wang Y, Wang S, Ding M, Chen Z (2023). Excitatory somatostatin interneurons in the dentate gyrus drive a widespread seizure network in cortical dysplasia. Signal Transduct Target Ther.

