# Supplementary material for: Impaired GABAergic regulation and developmental immaturity in interneurons derived from the medial ganglionic eminence in the tuberous sclerosis complex
Source: Acta Neuropathol. 2024 May 7;147(1):80. doi: 10.1007/s00401-024-02737-7 (PMC11076412; doi:10.1007/s00401-024-02737-7)
Supplement: Supplementary file 1 — Supplementary file1 (DOCX 203 KB) [file 401_2024_2737_MOESM1_ESM.docx]

| **ID** | **PA Diagnosis** | **Gender** | **Onset epilepsy age (m/y)** | **Age at operation (m/y)** | **Gene** | **Cause of death** | **Brain area** | **Used for** |
| --- | --- | --- | --- | --- | --- | --- | --- | --- |
| 1 | Control | F | - | 2 m | - | \| Myocarditis \| \| --- \| \|  \| | Frontal cortex | snRNA-seq |
| 2 | Control | M | - | 15 | - | Cardiac arrhythmia | Frontal cortex | snRNA-seq, immunohistochemistry |
| 3 | Control | F | - | 2 | - | Respiratory failure | Frontal cortex | snRNA-seq, immunohistochemistry |
| 4 | Control | M | - | 13 | - | Pneumonia | Frontal cortex | snRNA-seq |
| 5 | Control | F | - | 17 | - | Pneumonia | Frontal cortex | snRNA-seq |
| 6 | Control | M | - | 10 | - | Myocarditis | Frontal cortex | snRNA-seq, immunohistochemistry |
| 7 | Control | M | - | 13 | - | Pneumonia | Temporal cortex | Oocytes experiments |
| 8 | Control | F | - | 39 | - | Pneumonia | Frontal cortex | Oocytes experiments |
| 9 | Control | F | - | 25 | - | Respiratory failure | Frontal cortex | Oocytes experiments |
| 10 | TSC | F | 3 m | 13 | TSC2 | - | Frontal cortex | snRNA-seq, immunohistochemistry, oocytes experiments |
| 11 | TSC | M | 4 m | 2 | TSC2 | - | Frontal cortex | snRNA-seq |
| 12 | TSC | M | 3 m | 2 | TSC2 | - | Frontal cortex | snRNA-seq |
| 13 | TSC | M | 1 m | 2 | TSC2 | - | Frontal cortex | snRNA-seq |
| 14 | TSC | M | 1 m | 5 | TSC2 | - | Frontal cortex | snRNA-seq, immunohistochemistry |
| 15 | TSC | M | 1.5 m | 8 m | TSC2 | - | Frontal cortex | snRNA-seq |
| 16 | TSC | F | 3 m | 8 | TSC2 | - | Frontal cortex | snRNA-seq |
| 17 | TSC | F | 8 y | 9 | TSC2 | - | Frontal cortex | snRNA-seq |
| 18 | TSC | F | 1 m | 17 | TSC2 | - | Frontal cortex | snRNA-seq, immunohistochemistry |
| 19 | TSC | M | 1 m | 3 | TSC2 | - | Frontal cortex | snRNA-seq |
| 20 | TSC | F | 1 m | 10 m | TSC2 | - | Frontal cortex | snRNA-seq |
| 21 | TSC | M | 6 y | 14 | TSC1 | - | Frontal cortex | Oocytes experiments |
| 22 | TSC | F | 16 y | 21 | TSC1 | - | Frontal cortex | Oocytes experiments |
| 23 | TSC | F | 1 m | 30 | TSC2 | - | Frontal cortex | Oocytes experiments |
| 24 | TSC | M | 5m | 35 | TSC2 | - | Frontal cortex | Oocytes experiments |

**Supplementary table 1. Clinical information of control and TSC cases.**

M: male, F: female; TSC: tuberous sclerosis complex; snRNA-seq: single-nuclei RNA sequencing

**Supplementary table 2. Cell type markers used in annotation of cell type clusters.**

| **Cell type** | **Marker genes** |
| --- | --- |
| Excitatory neurons | SLC17A7, SATB2, SLC17A6, NMDAR1 |
| Inhibitory neurons | GAD1, GAD2 |
| Astrocytes | GFAP, ALDH1L1, AQP4, GLT1, S100B |
| Microglia | PTPRC, ITGAM, P2RY12, TMEM119 |
| Oligodendrocytes | OLIG1, GLDN11, MOG, MBP |
| OPCs | PDGFRA, CSPG4 |


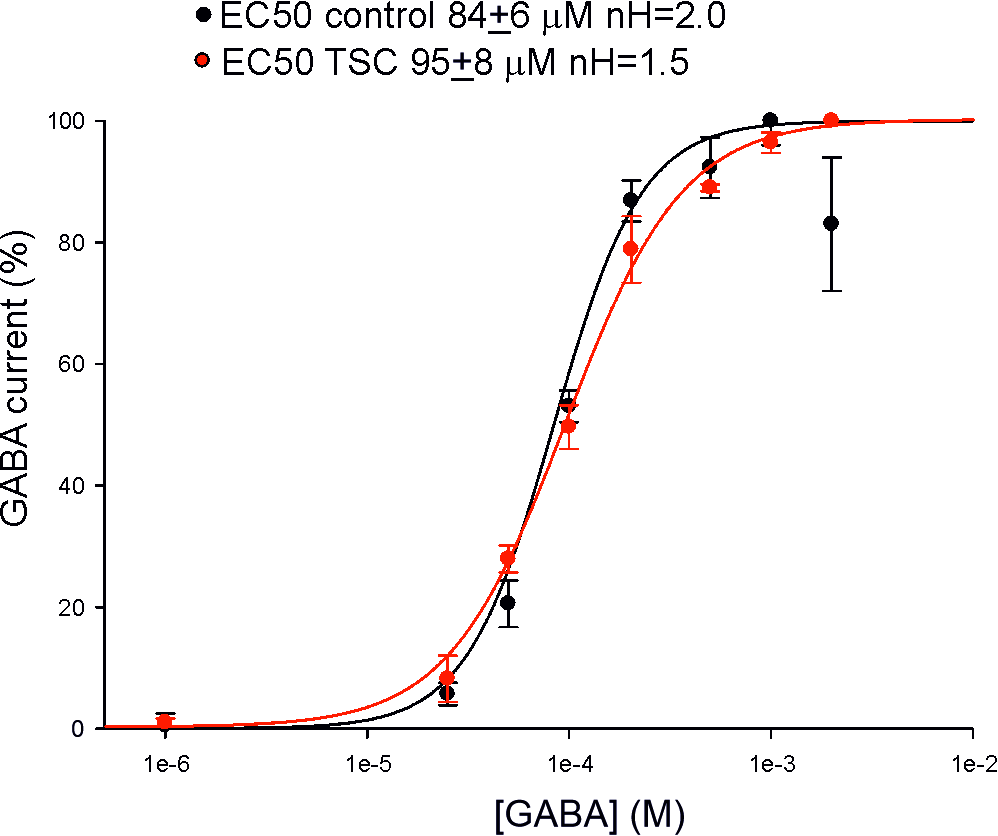


**Supplementary figure 1. GABA_A_R** **apparent affinity (EC_50_) in TSC tissues compared to control tissues**. The graph shows the amplitudes (as mean ± standard error of the mean [SEM]) of GABA responses recorded using different GABA concentrations, expressed as a percentage of the maximal current evoked current and best fitted by Hill curves. The EC_50_ values and nH were 95.0 ± 8.0 μmol/L and 1.5 ± 0.7 in oocytes injected with TSC membranes (●, 3 patients (10, 21, 24)) and 84.0 ± 6.0 μmol/L and 2.0 ± 0.2 in oocytes injected with control membranes (●, 3 patients); *P* > 0.05, unpaired *t* test).


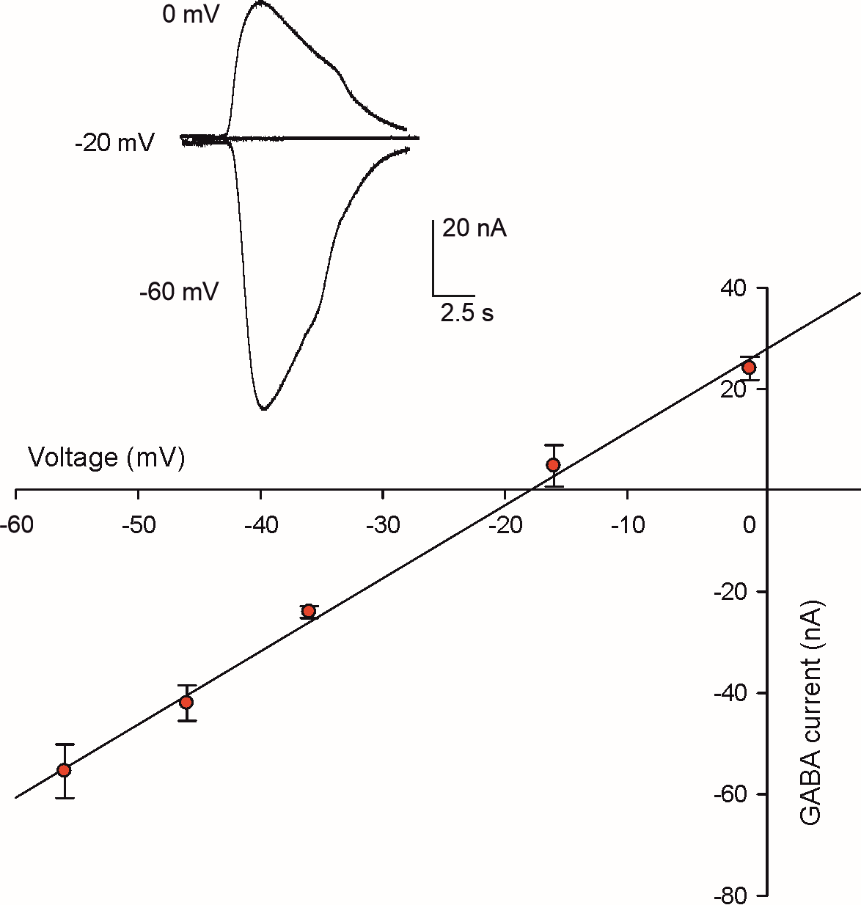


**Supplementary figure 2.**  **GABA reversal potential (E_GABA_) alteration in TSC.** The graph shows current‐voltage (I‐V) relationships from oocytes injected with membranes of TSC tissues. The points represent means ± standard error of the mean [SEM] of peak GABA currents normalized to I_max._ We recorded a mean E_GABA_ at −16.4 ± 0.5 mV (I_max_ = 55.4 ± 5.5 nA; n = 29, 3 TSC patients (21, 22, 23)). *Inset* shows sample currents from the same experiments at the holding potentials as indicated (in millivolts).
